# Anger management for people with mild to moderate learning disabilities: Study protocol for a multi-centre cluster randomized controlled trial of a manualized intervention delivered by day-service staff

**DOI:** 10.1186/1745-6215-12-36

**Published:** 2011-02-09

**Authors:** Paul Willner, Andrew Jahoda, John Rose, Biza Stenfert-Kroese, Kerenza Hood, Julia K Townson, Jacqueline Nuttall, David Gillespie, David Felce

**Affiliations:** 1 Directorate of Learning Disability Services, Abertawe Bro Morgannwg University Health Board, and Dept. of Psychology, Swansea University, UK; 2 Psychological Medicine, University of Glasgow, Gartnaval Royal Hospital, Glasgow, UK; 3 Psychology Department, University of Birmingham, UK; 4 South East Wales Trials Unit, Department of Primary Care & Public Health, School of Medicine, Cardiff University, Cardiff, UK; 5 Psychological Medicine, School of Medicine, Cardiff University, Cardiff, UK

## Abstract

**Background:**

Cognitive behaviour therapy (CBT) is the treatment of choice for common mental health problems, but this approach has only recently been adapted for people with learning disabilities, and there is a limited evidence base for the use of CBT with this client group. Anger treatment is the one area where there exists a reasonable number of small controlled trials. This study will evaluate the effectiveness of a manualized 12-week CBT intervention for anger. The intervention will be delivered by staff working in the day services that the participants attend, following training to act as 'lay therapists' by a Clinical Psychologist, who will also provide supervision.

**Methods/Design:**

This is a multi-centre cluster randomized controlled trial of a group intervention versus a 'support as usual' waiting-list control group, with randomization at the level of the group. Outcomes will be assessed at the end of the intervention and again 6-months later. After completion of the 6-month follow-up assessments, the intervention will also be delivered to the waiting-list groups. The study will include a range of anger/aggression and mental health measures, some of which will be completed by service users and also by their day service key-workers and by home carers. Qualitative data will be collected to assess the impact of the intervention on participants, lay therapists, and services, and the study will also include a service-utilization cost and consequences analysis.

**Discussion:**

This will be the first trial to investigate formally how effectively staff working in services providing day activities for people with learning disabilities are able to use a therapy manual to deliver a CBT based anger management intervention, following brief training by a Clinical Psychologist. The demonstration that service staff can successfully deliver anger management to people with learning disabilities, by widening the pool of potential therapists, would have very significant benefits in relation to the current policy of improving access to psychological therapies, in addition to addressing more effectively an important and often unmet need of this vulnerable client group. The economic analysis will identify the direct and indirect costs (and/or savings) of the intervention and consider these in relation to the range of observed effects. The qualitative analyses will enhance the interpretation of the quantitative data, and if the study shows positive results, will inform the roll-out of the intervention to the wider community.

**Trial registration:**

ISRCTN: ISRCTN37509773

## Background

Cognitive behaviour therapy (CBT) is the treatment of choice for common mental health problems [1], and in the UK is recommended by the National Institute for Clinical Excellence (NICE) for this purpose. Widening access to CBT for people with mental health problems is seen as a major policy priority: the UK Department of Health has recently allocated £170 million to train 3600 CBT therapists in England through the Increasing Access to Psychological Therapies (IAPT) programme [2]. However, people with learning disabilities are unlikely to benefit from this development, as their particular needs have not been identified within the current policy and the necessary research on effectiveness for this population is still at a rudimentary stage. The diagnostic term 'learning disability' is used in the UK to refer to people who meet the World Health Organization definition of 'intellectual disability' ("significant impairments of both intellectual and functional ability, with age of onset before adulthood"), and is equivalent to the term 'mental retardation' as used until recently in the USA [3]. It is only recently that CBT has been adapted for people with learning disabilities, and the evidence of its effectiveness in this population consists largely of case studies and case series. There is a relatively large case-study literature describing successful outcomes for CBT in a variety of mental disorders [4-7]. However, the evidence from controlled trials is sparse.

The most developed evidence base is in relation to anger. Anger is a frequent problem for many people with learning disabilities. Although anger can exist without being expressed aggressively, anger in people with learning disabilities is typically associated with verbal and/or physical aggression [8]. Aggression is the main reason for an adult with a learning disability to be regarded as having severe challenging behaviour [9] and to be referred for resource intensive intervention [10]. Left unchecked, aggression resulting from uncontrolled anger can lead to serious consequences, which include exclusion from services, breakdown of residential placements, and in extreme cases, involvement with the criminal justice system [11-13]. Aggressive behaviour can also have an impact on the psychological well-being of staff [14] and the quality of care they provide [15]. Community services supporting adults with learning disabilities receive numerous referrals for anger problems: prevalence estimates for problem anger in the general population of people with learning disabilities vary between 11 and 27% [16]. A review of recent studies of aggressive challenging behaviour reported that over half of the population of people with learning disabilities display some form of aggression [17], and anger is highly prevalent in people labelled as having challenging behaviour: for example, Lindsay and Law (1999) reported that 60% of clients referred to a community service for people with learning disabilities and challenging or offending behaviours presented with clinically significant anger problems [18].

Challenging behaviour has traditionally been managed pharmacologically or behaviourally [19,20]. However, following the demonstration that a CBT anger management intervention can decrease anger and aggression [21-23], the past 20 years has seen an increasing take-up of anger management as the first-line approach to these problems. With the exception of two small controlled trials in depression [24,25], anger is the only psychological presentation in which controlled trials have been used to evaluate CBT interventions for people with learning disabilities. Several phase 2 trials have now been published in which CBT for anger has been compared with a waiting-list control condition. These include seven studies of anger management groups in community settings and one series of studies of individual treatment in a forensic setting [26], as well as a single study of individual therapy in a community setting [16]. However, these typically have been relatively small studies, and have not used fully randomized allocation to treatment [26,27]. The published studies are fully consistent in reporting that anger interventions are effective in helping people with learning disabilities to manage their anger better, and that treatment gains are maintained at three or six-month follow up [26]. There is also evidence that treatment gains generalize across settings. There is little information as to which are the crucial components of the intervention. However, one recent study reported a significant correlation between decreased anger reactivity and increased usage of anger coping skills, thus providing some evidence that the specific psycho-educational content of the anger management curriculum is intrinsic to its effectiveness [28].

A recent Cochrane review of interventions for aggressive behaviour in people with learning disabilities [27] identified only four studies suitable for inclusion, including one study of group-based CBT for anger [29] and one study of individual CBT for anger [30]. The review concluded that: *"The existing evidence on the efficacy of cognitive behavioural and behavioural interventions on outwardly directed aggression in children and adults with learning disabilities is scant. There is a paucity of methodologically sound clinical trials. Given the impact of such behaviours on the affected individual, his or her carers and on service providers, effective interventions are essential. It is also important to investigate cost efficacy of treatment models against existing treatments. We recommend that randomised controlled trials of sufficient power are carried out using primary outcomes of reduction in outward directed aggression, improvement in quality of life and cost efficacy as measured by standardised scales" *[27].

This trial will evaluate the effectiveness, compared to normal care, of a manualized anger management intervention, delivered to people with mild to moderate learning disabilities in a service setting and by service staff, in reducing levels of reported anger.

## Methods/Design

### Ethical and governance approval

Multi-centre approval has been granted by South East Wales Research Ethics Committee (**09/WSE03/41**). R&D approval has been granted in all regions, with additional participation identification centre approval where required. ISRCTN reference number is ISRCTN 37509773.

### Design

**Figure 1:**
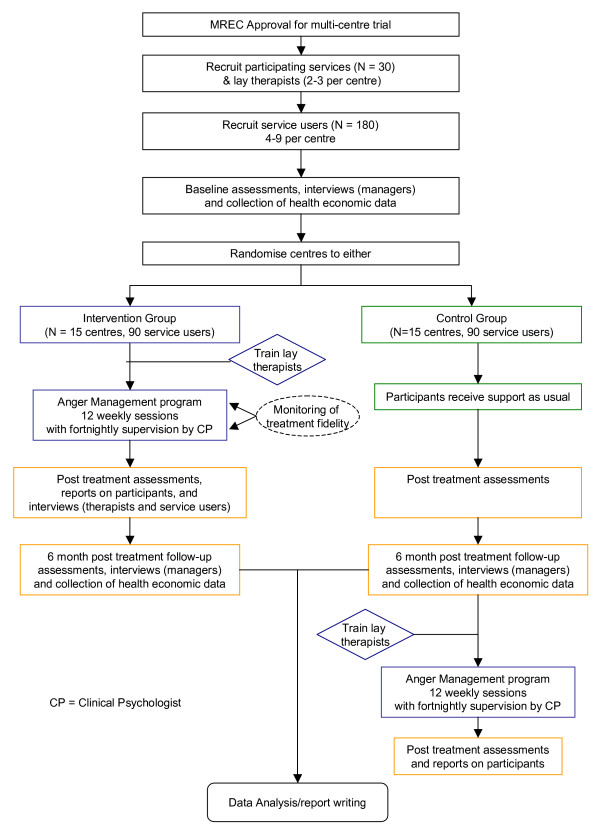
**Trial Design**.

This is a multi centre cluster randomized controlled trial of a manualized anger management group intervention versus a 'support as usual' waiting-list control group, with randomization of the group rather than the individual. A cluster randomized design, with one group per participating centre, was adopted to avoid the contamination between arms that would result if intervention and control groups were recruited within the same centre. The trial design is summarized in Figure 1

### Sample size

Published studies of anger management in people with learning disabilities typically report large effect sizes. As service staff might be less effective therapists than psychologists, we aim to detect a medium-sized effect (d = 0.57). This estimate is a conservative 40% of the effect size (d = 1.35) observed in an earlier controlled trial using the same endpoint [31].

To achieve significance at p < 0.05 with 80% power will require two groups of n = 72 (allowing for ICC = 0.11). As there is no basis for estimating an ICC in the present context, we have used a value just above the range of ICC values reported in a recent systematic review [32], which varied between 0.01 and 0.1. This allows for the level of clustering that we would expect to see between participants naturally. As this is a group-based intervention, the effect in the intervention arm may well be to increase the degree of clustering. The analysis of the study will allow for this, but the sample has only been inflated to allow for the underlying level of clustering of service users within services rather than the component that relates to intervention effect.

To arrive at the target of 72 participants in each arm of the trial, a single group of 4-9 service users (average = 6) will be recruited in each of 30 participating centres. This total of 180 participants allows for 20% loss to follow-up, which is a conservative estimate: no drop-out was observed in two earlier studies conducted in day-service settings [31,28] and this is a relatively static population.

### Service and participant recruitment

Thirty services providing day activities for people with mild to moderate learning disabilities will be recruited, on the basis that they report significant anger control problems among some of their service users. Within the current mixed-economy of care such services may be run by statutory or independent sector providers, and may vary in their mode of operation from traditional day centres to individualized community-based activity programmes. In order to recruit a sufficient number of centres within the time frame of the project, it is being implemented in three different regions, one in Wales, one in England and one in Scotland. In each region, 10 services will be identified, of which 5 will be randomly allocated to the intervention group and 5 to the control group. Within each group a minimum of 4 and a maximum of 9 service users will be recruited, which will mean a total of approximately 180 service users recruited on to the trial.

Potential service users are eligible for the trial if they meet all of the inclusion criteria and none of the exclusion criteria (Table 1).

**Table 1:** Inclusion and Exclusion Criteria

Inclusion criteria (Service Users)	Exclusion criteria (Service Users)
1. An adult attending a service for people with mild to moderate learning disabilities	1. Attending the service for a reason other than a diagnosed learning disability
2. Identified by service staff as having problems in managing their anger	2. Currently receiving psychological treatment for anger or aggression
3. Wishing to learn to improve their anger management	3. Urgently requiring referral to a Clinical Psychologist for individual treatment of anger or aggression
4. Able to provide informed consent	4. Experiencing circumstances which indicate that a Protection of Vulnerable Adults (POVA) procedure should be initiated
5. Able to complete the assessments	5. If for any other reason the supervising Clinical Psychologist makes a clinical judgement that participation in the group would be counter-indicated

**Inclusion criteria (Services)**	**Exclusion criteria (Services)**

1. Reported anger control problems among at least four service users who meet individual inclusion criteria and want to participate	1. The service is already running an anger management programme similar to this one
2. Availability of at least two staff members willing to be trained as group leaders	2. There are no suitable facilities for group work
3. The service manager will provide written agreement to participate	

#### Other participants

For each service user a key worker, and where applicable, a home carer will also be recruited (not all service users will have a home carer depending upon their residential setting). In each participating centre, at least two (wherever possible, three) staff will be recruited to act as lay therapists. Staff will be nominated by their manager and selected on the basis of their motivation to take on this role and their openness to use a cognitive behavioural approach, without reference to formal qualifications. Service Managers will also be recruited from each participating centre.

### Randomisation

Randomisation will be performed using the method of minimisation [33,34]. Centres will be balanced on their service users' average baseline self-reported Provocation Index scores, the number of service users recruited and the average number of hours a week spent by the service user with at least one trainer outside of sessions. A random component, set at 80%, will be used alongside the minimisation procedure.

Centres will be recruited, and baseline data will have been collected on all participating service users (of a particular centre), before randomisation of that centre takes place.

Centres will be randomised using an automated service provided by SEWTU. Selection bias can be a problem in cluster-randomised trials if participants are recruited after cluster allocation has been revealed [35]. Therefore, all services and participants will be recruited and assessed prior to randomisation. In order to maintain engagement in services randomised to the control group, training is offered at the end of the study, and both groups will receive funding to cover the costs of replacing the staff who act as lay therapists. The research assistants who undertake the assessment of the outcomes will not have any involvement in the delivery of the intervention. As far as is possible they will be blinded to the group allocation of the service, although during direct interaction with the service user the group allocation may become apparent. Where this occurs it will be recorded.

### Trial procedures

#### Intervention

Participants will receive a manualized CBT intervention [28], consisting of 12 weekly psycho-educational group sessions supplemented by 'homework'. Before the start of the intervention, a Clinical Psychologist will provide the lay therapists with 2-3 training sessions, covering the principles of anger management and use of the therapy manual, followed by fortnightly supervision during the intervention. Additional training sessions could be provided, at the discretion of the trainer. Staff training will follow a training manual developed within the project for this purpose.

Topics addressed over sessions include: the triggers that evoke anger; physiological and behavioural components of anger; behavioural and cognitive strategies to avoid the build-up of anger and for coping with anger-provoking situations; and acceptable ways of displaying anger (assertiveness). Presentation relies heavily on brainstorming (e.g. "What makes us angry?") and role-play. After the first session, about a third of each session, is devoted to discussion by facilitators and group members of one or two participants' experiences, focussing primarily on problem solving around ways in which situations might have been handled differently to produce a better outcome. In addition to simplifying the language used in sessions, we avoid wherever possible the use of written materials, in favour of pictorial representations. Towards the end of every session, participants are asked to undertake a homework assignment, which consists of working with a staff member to complete a functional analysis ('hassle log'), of a situation in which they have been angered that week, which is described, analysed and evaluated, using a pictorial work-book. At the end of the intervention, reports are provided on each of the participants, and recommendations are made for further input by staff to maintain and increase treatment gains. A version of each report will also be produced in a format accessible to the service user.

#### Frequency and duration of follow up

All participants will be followed up post intervention, ie 16 weeks post randomisation and again 6 months later. The 16-week time point is chosen to allow two weeks before the start of the 12-week intervention for staff training, and two weeks to take account of likely delays due to centre closures or staff absences. After the 6-month follow-up the intervention will be delivered to the control group, followed by further post-intervention assessments (Figure 1): these data will be used in secondary analysis that are not described here.

A contractual agreement has been negotiated with participating services. Consent will be sought from five types of participants: the service users themselves, their key-workers and home carers, lay therapists and service managers. Written consent is taken from the service managers, lay therapists, key-workers and home carers, using consent forms and procedures that comply with standard Research Ethics Committee guidelines.

**Figure 2:**
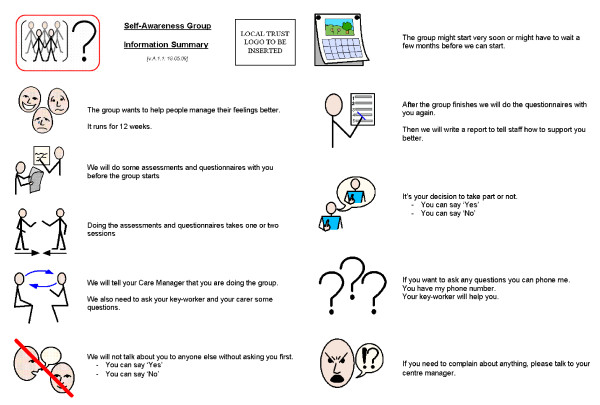
**Accessible information sheet**.

For service users, a more accessible consent procedure is used:

(i) The trial is explained verbally in simple terms, using a standard script written in accessible language, and checking frequently for understanding. It is important, when working with people with intellectual disabilities, to restrict the amount of information presented, so as to avoid information overload; therefore, the information script contains less information than might be usual with more able participants.

(ii) In addition to the general information sheet that is provided to all participants, service users are also given a simplified accessible information sheet, to take home and read in their own time and at their own speed, with support from carers (Figure 2)

(iii) At least two days are given to consider and ask questions of researchers or carers: long delays could be counter-productive in this client group.

(iv) The explanation is repeated in a second meeting.

(v) Consent is recorded by the service user checking and initialling a set of tick boxes and signing the consent form.

(vi) In order to assure that the service user has been properly informed, without coercion, the whole process is witnessed and signed off by a staff member who is independent of the research team.

For therapists and service users selected for interview after the end of the intervention (see below), a separate consent will be taken at the time, using the same procedures as above.

### Outcome measures

#### Quantitative assessments

**Figure 3:**
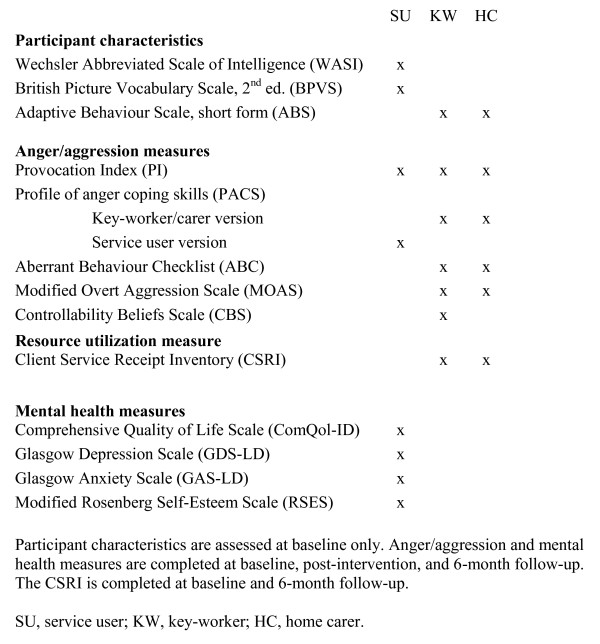
**Overview of quantitative assessments**.

An overview of the quantitative assessments is shown in Figure 3.

##### Participant Characteristics

Intellectual and receptive language abilities will be assessed at baseline using the Wechsler Abbreviated Scale of Intelligence (WASI) and the British Picture Vocabulary Scale, 3rd edition (BPVS), respectively. Adaptive behaviour will be assessed using the short form of the Adaptive Behavior Scale [36], which is completed by the service-user's key-worker or home carer.

##### Quantitative Outcome Evaluation

Quantitative measures will be administered before and after treatment and at 6-months after the end of treatment. The researchers undertaking these outcome assessments will not have any involvement in training or supervision of the therapists. They will undertake fidelity monitoring, but they will do this in a region other than the one where they conduct assessments.

##### Primary Outcome measure

The main outcome measure will be the Provocation Index (PI) as completed by the service-user, at follow-up. The PI is a direct measure of felt response to defined situations that may provoke anger that has frequently been used with this service-user group [37,8]. It consists of a list of 25 different situations that can evoke anger, each of which is rated on a four-point scale for the amount of anger that it would evoke. Scores on this measure have been shown to correlate with staff-reported levels of aggression [8].

##### Secondary Outcome measures

Assessment will also involve completion of the PI by a key-worker [31,28]. For this and other measures, in the event that a service-user's key-worker is involved in the trial as a lay therapist, then the measure will be completed by another staff member.

Aggression will be assessed by key-worker report using the Irritability domain items of the Aberrant Behavior Checklist (ABC) [38] and the Modified Overt Aggression Scale (MOAS) [39]. Both assessments have been either designed or validated for assessment of people with learning disabilities, and were used to assess behaviour in a recent RCT of pharmacological treatment of aggressive challenging behaviour in adults with a learning disability [40]. Key-workers' attributions in respect of challenging behaviour will be measured by the Controllability Beliefs Scale (CBS) [41].

The Profile of Anger Coping Skills (PACS) [31,28] will be completed by both service-user and key-worker to assess the development of alternative, more functional coping skills.

Mental health will be assessed by using the Glasgow Depression and Anxiety Scales, which are established measures of depression and anxiety among people with a learning disability [42,43], and an adaptation of the Rosenberg Self-Esteem Scale for people with a learning disability [44]. Self-reported quality of life will be assessed by using the Comprehensive Quality of Life Scale - Intellectual Disability (ComQoL-ID) [45]. While it is predicted that successful acquisition of anger control skills might improve mental health and quality of life, these measures will also serve to detect adverse effects of treatment.

So as to assess generalization across settings, the anger, aggression and coping skills measures (PI, ABC, MOAS, PACS) will also be completed with service users' home carers, where appropriate.

#### Qualitative Assessments

One service user from each group (N = 15) will be interviewed after the intervention to gain an understanding of their experiences of participating in CBT. Service users will be randomly selected from a "short list" of those participants who are considered to have sufficient expressive language ability to be interviewed. This part of the research is not hypothesis driven but aims to gain an 'insider's perspective' from which a theoretical framework regarding the subjective experiences of service users can be developed.

The therapist who has been most active in terms of running each group (N = 15) will be interviewed post-intervention in order to investigate their experiences of learning and applying new therapeutic skills as cognitive behavioural therapists, as well as their impressions of the 'climate' within the group and the impact of the group on the wider service.

Both service user and therapist interviews will be conducted according to a semi-structured interview schedule, containing questions that encourage the participants to focus on 'personal meaning' and making sense of their experiences of the therapeutic process.

A related, but separate, part of the qualitative evaluation aims to gain an understanding of service policies and practices for service users who express anger inappropriately. This will be accomplished by interviewing the managers of all of the services in the intervention arm (N = 15), before the intervention and at follow-up.

### Health-economic evaluation

The economic analysis will be in the form of a service-utilization cost and consequences analysis. The costing will be undertaken as follows;

1) All resources used in delivering the intervention will be recorded prospectively and valued using standard methods [46] with unit costs provided by the study sites. Resource inputs would include:

(i) Time input of (a) the applicants to train/supervise the clinical psychologists implementing and supervising the intervention, (b) the clinical psychologists in training and supervising the day service lay therapists running the groups, (c) the day service lay therapists in running the groups, (d) administrative/secretarial staff attributable to the intervention.

(ii) Travel costs attributable to the intervention.

(iii) Consumable costs attributable to the intervention (e.g. production of manuals).

2) All other resources used by study participants at the intervention sites, and all resources used by study participants at control sites will be monitored prospectively, using recording logs overseen by the research team. These will be valued using standard methods with unit costs provided by the study sites.

3) All relevant resource use, apart from that at the study sites, will be collected at baseline and at follow-up using the Client Service Receipt Inventory (CSRI). The CSRI is a validated tool to measure total package resource use and has been used in evaluations involving service users with psychiatric problems and service users with learning disabilities [47,48]. It records items such as contacts with community-based primary care, other health or social services, educational services, outpatient and inpatient attendances, etc. Unit costs for most of these are available [49]. Information will be collected from service-users' key-workers and/or home carers for the three-month period immediately preceding data collection.

In relation to (2) and (3) above, we propose specifically to investigate staff allocation within the provided services. Service managers will be interviewed to ascertain the staff-to-service-user ratios allocated to participants at baseline and at follow-up. As the national compendium cost figures give a breakdown of costs for day and residential services in which staff costs are identified, we will be able to adjust the staff cost element to reflect any changes in staff deployment between baseline and follow-up reported. Staff costs are the largest component of costs within day and residential services and arguably most likely to change as a result of treatment (e.g., in comparison with food, lighting, heating, estate, administrative and agency overhead costs).

### Process Evaluation

An instrument to measure the fidelity of the intervention has been developed specifically for this trial. It measures therapists' adherence to the treatment manual and to the principles of cognitive behavioural therapy. In each centre, two sessions will be observed and recorded, by two members of the research team who have no other contact with that centre. The first observation will take place between week 3 and week 6 of the group and the second between week 7 and 10, with at least 3 weeks between the two observations.

## Data analysis

### Quantitative outcomes

The primary analysis will be intention to treat and will compare the mean self-reported PI between the two groups using a two level linear regression model, with participants at level 1 and centres at level 2, with baseline levels of the PI as a covariate. Secondary outcomes will be analysed similarly. Variables will be transformed prior to analysis if necessary to fulfil assumptions of normality.

Formal subgroup analysis of those who are above a threshold of self-reported PI of 1.0 at baseline, and those who meet formal criteria for a diagnosis of 'learning disability', will be undertaken through the fitting of interaction terms to the primary model. Other exploratory analysis will assess whether or not the effect of the intervention differs in different service settings (statutory/independent) and by intellectual and language ability.

A complier adjusted causal effect (CACE) will be estimated using a multi-level mixture analysis [50] to assess the impact of non-compliance with the intervention on the effect shown. A complier will be taken as someone who has attended at least two thirds of the sessions (8 of 12). None of the control group will be able to access the intervention until after follow-up is completed.

The associations between self and key worker/home carer reports will be assessed and compared between intervention and control groups. The association between anger coping skills and outcomes such as provocation, mental health and QoL will also be assessed. Secondary analyses to identify factors predictive of outcomes will be conducted using regression methods, based on the total cohort of N = 180, following delivery of the intervention to the control groups.

### Qualitative outcomes

Service user interviews will be analyzed using Interpretative Phenomenological Analysis (IPA). IPA attempts to reduce the complexity of experiential data through vigorous and systematic analysis in a transparent and plausible manner [51]. It has a specific psychological focus and is suitable for data collected from less articulate/forthcoming participants. The focus of the therapist interviews is on the therapists' personal, subjective experiences and therefore IPA will again be utilised as the most appropriate qualitative analysis.

As the focus of the service manager demands a structured, factual line of enquiry, Thematic Analysis (TA) [52] will be used to categorise participants' responses into themes and sub-themes. Responses will be grouped according to each of the questions posed during the structured interview and will be analysed as such, in order to establish common themes and differences within and between services before and after the intervention. A particular focus of this part of the evaluation will be the perceived influence that the CBT trials have had on professional practice within each of the services.

Both of the qualitative evaluations (IPA and TA) will be subjected to a credibility check, by presenting relevant participants with a summary account of the findings in order to establish whether the analyses have produced an account which is credible and comprehensible to its informants.

### Health economic outcomes

The analysis is directed at addressing three inter-related questions: (a) the extent to which there is added resource usage above support as usual arising from providing the intervention, (b) the extent to which service package costs at follow-up differ from those at baseline, and (c) if both a and b reveal differences in hypothesised directions, the extent to which additional costs might be offset by subsequent savings in ongoing support. (Clearly, it will be redundant to conduct this third stage if the intervention is cheaper to provide than support as usual and results in reduced service package costs or if the intervention is more expensive to provide than support as usual and results in greater service package costs.)

In principle, the cost data will not be treated differently from other data in the analysis. Cost data are frequently skewed. Tests for normality will be applied. If the distribution of data is shown to be non-normal, non-parametric bootstrapping methods will be used to test for differences in costs between groups [53]. Bootstrapping produces a confidence interval for the difference between the means and significance is judged by whether the confidence interval contains zero. Specifically, if the confidence interval does not contain zero, it is assumed that the difference between the means is significant. Bootstrapping does not produce a conventional test statistic by which the level of significance can be judged. Confirmatory, post-hoc, non-parametric testing can be performed to obtain conventional significance levels.

We are primarily reliant on the research to establish associations between the intervention and outcomes including cost. However, we will also attempt to interpret cost changes found. For example, were there to be changes in staff allocation post treatment, we would ask service managers to say what underlay this change, or, if a person moved from living in the family home to an out-of-family placement, we would find out the reason (e.g., did it relate to the person's behaviour or had family capacity to provide care diminished).

## Discussion

This will be the first multi-centre trial to investigate formally how effectively staff working in services providing day activities for people with learning disabilities are able to use a therapy manual to deliver a CBT-based anger management intervention, following brief training by a Clinical Psychologist. The study incorporates a wider range of outcome measures than previous studies, and includes an analysis of the cost consequences of delivering the intervention. The demonstration that service staff can successfully deliver anger management to people with learning disabilities would have very significant benefits in relation to the current policy of improving access to psychological therapies, by widening the pool of potential therapists, in addition to addressing more effectively an important and often unmet need of this vulnerable client group. Some scepticism has been expressed about whether it is feasible to undertake randomised controlled trials of psychological interventions for people with learning disabilities [54]. The successful implementation of this RCT would serve to allay these doubts and encourage further research to strengthen the evidence base for interventions to support this population. We hope also to identify factors relating to characteristics of participants or settings that are associated with differential outcomes. The study will also pilot a fidelity-monitoring instrument that is not anger specific, but rather, has been designed to be applicable to any CBT-based group therapy for people with learning disabilities.

Felce et al. (2003) found that 26% of the variance in staff costs per person in residential services was associated with scores on a challenging behaviour measure (the Aberrant Behavior Checklist) that we are using as one of our outcome measures [55]. The service-utilization cost and consequences analysis will determine the extent to which delivering the intervention incurs resource inputs over and above support as usual, and whether successful reduction of anger and aggression is associated with any change in subsequent resource use. We rejected a cost utility approach because we believe that the utility-based health state measures such as EQ-5D required for such analyses would not be sensitive to the effects anticipated from the intervention. We rejected a cost effectiveness approach partly because of the multiple objectives of the intervention (e.g. aggression, controllable beliefs, coping, self esteem), which cost effectiveness analysis cannot handle, and partly because the primary outcome measure, the provocation index (PI), is not an effect that features in economic analyses of related interventions. Unless our intervention is shown to be dominant (more effective and less costly than usual care) then an incremental cost effectiveness ratio in terms of extra cost per unit PI would be of little value in informing policy. The proposed cost and consequences analysis is, strictly, not a technique of economic evaluation as it cannot provide a definitive answer to questions of either allocative or technical efficiency. It does however identify the direct and indirect costs (and/or savings) of the intervention and considers these in relation to the range of observed effects.

We anticipate that the qualitative data will enhance the quantitative analyses in four distinct ways. First, as with any well-designed mixed-methods study, we will aim to generate a productive interaction between the quantitative and qualitative analyses: exploratory quantitative analysis will be undertaken to explore possible inter-relationships between factors identified in the qualitative analysis; similarly the qualitative data will be reviewed to explore evidence in relation to findings that emerge from the quantitative modelling. Second, while the quantitative data will provide answers to the question of the effectiveness of the intervention, they will not provide insights into the process or mechanisms of change. This information will, however, emerge from an account of the participants' (both service users and staff) experience of the groups and their understanding of the intervention. The qualitative findings will also influence the interpretation of the outcome data by indicating the personal salience (as distinct from the statistically significance), for clients or those affected by their behaviour, of any changes that are found. And if there are negative results, the qualitative data, alongside the assessment of the fidelity of intervention delivery, may help to explain them. Third, interviewing participants and staff may identify unanticipated outcomes of the group, either positive or negative, and barriers to change. (For example, in a study of anger management by adolescents, qualitative analysis identified some clinically important but unanticipated moderating effects of participants' ages on outcomes, which were confirmed in a reanalysis of the quantitative data: [56].) Finally, if the study shows positive results, the qualitative data, including the impact of the intervention on the culture within day services, will inform the roll-out of the intervention to the wider community.

## Competing interests

The authors declare that they have no competing interests.

## Authors' contributions

PW is the Principal Investigator and led the study design and funding application, and the writing of this manuscript. AJ and JR are clinical psychologists who contributed to the study design and funding application, particularly in relation to the clinical intervention and quantitative outcome measures. BSK is a clinical psychologist who contributed to the study design and funding application, particularly in relation to the qualitative analyses. DF is a health services researcher who contributed to the study design and funding application, particularly in relation to the health economic evaluation. JN is the senior trial manager and JT is the trial manager and both contributed to the study design, management and analysis plan. DG is the trial statistician and contributed to the study design and analysis plan. KH is the senior statistician and contributed to the study design and analysis plan, and to the funding application and is the responsible individual to the sponsor. All authors contributed to, read and approved the final version of the manuscript.

## References

[B1] Roth A Fonagy P What works for whom? A critical review of psychotherapy research 2004 2 Guilford Press, New York

[B2] DoH/CSIP Choices in Mental Health: Improving Access to Psychological Therapies 2007 http://www.dh.gov.uk

[B3] British Psychological Society Learning Disability: Definitions and Contexts 2000 British Psychological Society, Leicester

[B4] Lindsay WR Cognitive therapy Psychologist 1999 12 238 241

[B5] Hatton C Psychosocial interventions for adults with intellectual disabilities and mental health problems: A review J Ment Health 2002 11 357 373 10.1080/09638230020023732

[B6] Willner P Readiness for cognitive therapy in people with intellectual disabilities J App Res Intellect Disabil 2005 19 5 16 10.1111/j.1468-3148.2005.00280.x

[B7] Taylor JL Lindsay WR Willner P CBT for people with intellectual disabilities: Emerging evidence, cognitive ability and IQ effects Behavioural and Cognitive Psychotherapy 2008 36 723 734 10.1017/S1352465808004906

[B8] Taylor JL Novaco RW Anger Treatment for People with Developmental Disabilities: A Theory, Evidence and Manual-Based Approach 2005 Wiley, Chichester

[B9] Allen D Felce D Bouras N Service responses to challenging behaviour Psychiatric and behavioural disorders in developmental disabilities 1999 Cambridge: Cambridge University Press

[B10] Lowe K Felce D Blackman D People with learning disabilities and challenging behaviour: the characteristics of those referred and not referred to specialist teams Psychol Med 1995 25 595 603 10.1017/S003329170003350X 7480439

[B11] Allen D Lowe K Brophy S Moore K Predictors, costs and characteristics of out of area placements for people with intellectual disability and challenging behaviour J Intellect Disabil Res 2007 51 409 416 10.1111/j.1365-2788.2006.00877.x 17493024

[B12] Emerson E Robertson J Dorr H Russell P Spencer K Davies I Felce D Allen D Churchill J Rose S Maguire S Hatton C Madden P Mills R McIntosh B Congdon D Commissioning Person Centred, Cost Effective, Local Support for People with Learning Difficulties 2008 London: Social Care Institute for Excellence

[B13] Mental Health Foundation Boiling Point 2008 http://www.mentalhealth.org.uk

[B14] Jenkins R Rose J Lovell C Psychological well-being of staff working with people who have challenging behaviour J Intellect Disabil Res 1997 41 502 511 10.1111/j.1365-2788.1997.tb00743.x 9430055

[B15] Rose J Jones F Fletcher B Investigating the relationship between stress and worker behaviour J Intellect Disabil Res 1998 42 163 172 10.1046/j.1365-2788.1998.00115.x 9617700

[B16] Rose J Dodd L Rose N Individual cognitive behavioural intervention for anger J Ment Health Res Intellect Disabil 2008 1 97 108 10.1080/19315860801988368

[B17] Benson BA Brooks W Aggressive challenging behaviour and intellectual disability Curr Opin Psych 2008 21 454 458 10.1097/YCO.0b013e328306a090 18650686

[B18] Lindsay WR Law J Outcome evaluation of 161 people with learning disabilities in Tayside who have offending or challenging behaviour, 1999 Paper presented to British Association for Behavioural and Cognitive Psychotherapy, 27th Annual Conference 1999 Bristol

[B19] Didden R Duker P Korzilius H Meta-analytic study on treatment effectivesness for problem behaviours in individuals who have mental retardation Am J Ment Retard 1999 101 387 399 9017085

[B20] Matson J Bamburg J Mayville E Pinkston J Bielecki J Kuhn D Psychopharmacology and mental retardation: A ten year review (1990-1999) Res Dev Disabil 2000 21 263 296 10.1016/S0891-4222(00)00042-1 10983783

[B21] Benson BA Rice CJ Miranti SV Effects of anger management training with mentally retarded adults in group treatment J Consult Clin Psychol 1986 54 728 279 10.1037/0022-006X.54.5.728 3534033

[B22] Benson B Teaching anger management to persons with mental retardation 1992 Champaign, IL: International Diagnostic System, Inc

[B23] Benson B Bouras N Anger management training: A self control program for people with mild mental retardation Mental Health in Mental Retardation 1994 Cambridge, UK: Cambridge University Press 224 232

[B24] McCabe MP McGillivray JA Newton DC Effectiveness of treatment programmes for depression among adults with mild/moderate intellectual disability J Intellect Disabil Res 2006 50 239 247 10.1111/j.1365-2788.2005.00772.x 16507028

[B25] McGillivray JA McCabe MP Kershaw MM Depression in people with intellectual disability: An evaluation of a staff-administered treatment program Res Dev Disabil 2008 29 524 536 10.1016/j.ridd.2007.09.005 17981010

[B26] Willner P CBT for people with intellectual disabilities: Focus on anger Adv Ment Health Learn Disabil 2007 1 14 21

[B27] Hassiotis I Hall I Behavioural and cognitive-behavioural interventions for outwardly-directed aggressive behaviour in people with learning disabilities Cochrane Database Syst Rev 2008 CD003406 18677776 10.1002/14651858.CD003406.pub3

[B28] Willner P Tomlinson S Generalization of anger-coping skills from day-service to residential settings Journal of Applied Research in Intellectual Disabilities 2007 20 553 562 10.1111/j.1468-3148.2007.00366.x

[B29] Willner P Jones J Tams R Green G A randomised controlled trial of the efficacy of a cognitive-behavioural anger management group for adults with learning disabilities J App Res Intellect Disabil 2002 15 224 235 10.1046/j.1468-3148.2002.00121.x

[B30] Taylor JL Novaco RW Gillmer BT Robertson A Thorne I Individual cognitive-behavioural anger treatment for people with mild-borderline intellectual disabilities and histories of aggression: A controlled trial Brit J Clin Psychol 2005 44 367 382 10.1348/014466505X29990 16238883

[B31] Willner P Brace N Phillips J Assessment of anger coping skills in individuals with intellectual disabilities J Intellect Disabil Res 2005 49 329 339 10.1111/j.1365-2788.2005.00668.x 15817050

[B32] Eldridge SM Ashby DS Feder GSR Rudnicka AR Ukoumunne OC Lessons for cluster randomized trials in the twenty-first century: a systematic review of trials in primary care Clin Trials 2004 1 80 90 10.1191/1740774504cn006rr 16281464

[B33] Scott NW McPherson GC Ramsay CR Campbell MK The method of minimization for allocation to clinical trials: a review Controlled Clinical Trials 2002 23 662 674 10.1016/S0197-2456(02)00242-8 12505244

[B34] Altman DG Bland JM Treatment allocation by minimisation BMJ 2005 330 843 10.1136/bmj.330.7495.843 15817555 PMC556084

[B35] Puffer S Torgerson D Watson J Evidence for risk of bias in cluster randomised trials: a review of recent trials published in three general medical journals British Medical Journal 2003 327 785 10.1136/bmj.327.7418.785 14525877 PMC214092

[B36] Hatton C Emerson E Robertson J Gregory N Kessissoglou S Perry J Felce D Lowe K Walsh PN Linehan C Hillery J The Adaptive Behavior Scale - Residential and Community (Part I) - Development of a short form Res Dev Disabil 2001 22 273 288 10.1016/S0891-4222(01)00072-5 11523952

[B37] Novaco RW Monahan J, Streadman HJ Anger as a risk factor for violence among the mentally disordered Violence and Disorder: Developments in Risk Assessment 1994 Chicago: University of Chicago Press 21 59

[B38] Aman MG Singh NN Stewart AW Field CJ The Aberrant Behavior Checklist: a behavior rating scale for the assessment of treatment effects Am J Ment Defic 1985 89 485 91 3993694

[B39] Oliver PC Crawford MJ Rao B Reece B Tyrer P Modified Overt Aggression Scale (MOAS) for people with intellectual disability and aggressive challenging behaviour: a reliability study J App Res Intellect Disabil 2007 20 368 72 10.1111/j.1468-3148.2006.00346.x

[B40] Tyrer P Oliver-Africano PC Ahmed Z Bouras N Cooray S Risperidone, haloperidol, and placebo in the treatment of aggressive challenging behaviour in patients with intellectual disability: a randomised controlled trial Lancet 2008 371 57 63 10.1016/S0140-6736(08)60072-0 18177776

[B41] Dagnan D Grant F McDonnell A Understanding challenging behaviour in older people: The development of the controllability beliefs scale Behav Cog Psychother 2004 32 1 6 10.1017/S1352465804001006

[B42] Cuthill FM Espie CA Cooper S-A Development and psychometric properties of the Glasgow Depression Scale for people with a learning disability: Individual and carer supplement versions British Journal of Psychiatry 2003 182 347 353 10.1192/bjp.182.4.347 12668412

[B43] Mindham J Espie CA Glasgow Anxiety Scale for people with an Intellectual Disability (GAS-ID): development and psychometric properties of a new measure for use with people with mild intellectual disability J Intellect Disabil Res 2003 47 22 30 10.1046/j.1365-2788.2003.00457.x 12558692

[B44] Dagnan D Sandhu S Social comparison, self-esteem and depression in people with intellectual disability Journal of Intellectual Disability Research 1999 43 372 379 10.1046/j.1365-2788.1999.043005372.x 10546961

[B45] Cummins RA The Comprehensive Quality of Life Scale: Intellectual Disability 1997 5 Melbourne, Australia: Deakin University

[B46] Drummond M Sculpher M Torrance G O'Brien B Stoddart G Methods for the Economic Evaluation of Programmes in Health Care 2005 3 Oxford University Press. Oxford

[B47] Beecham J Knapp MRJ Collecting and estimating costs The Economic Evaluation of Mental Health Care 1995 Aldershot: Arena

[B48] Beecham J Knapp MRJ Thornicroft GJ, Brewin CR, Wing JK Costing psychiatric interventions Measuring Mental Health Needs 1992 London: Gaskell

[B49] Curtis L Unit Costs of Health and Social Care 2008 2008 PSSRU

[B50] Jo B Asparouhov T Muthen B Intention-to-treat analysis in cluster randomised trials with non-compliance Statistics in Medicine 2008 27 5565 5577 10.1002/sim.3370 18623608 PMC2907896

[B51] Smith JA Beyond the divide between cognition and discourse: using interpretative phenomenological analysis in health psychology Psychology & Health 1996 1 39 54

[B52] Aronson J A pragmatic view of thematic analysis The Qualitative Report 1994 2 131 134 1994

[B53] Efron B Tibshirani R An Introduction to the Bootstrap 1993 New York: Chapman and Hall

[B54] Oliver PC Piachaud J Done J Regan A Cooray S Tyrer P Difficulties in conducting a randomised controlled trial of health service interventions in intellectual disability: implications for evidence-based practice J Intellect Disabil Res 2002 46 340 345 10.1046/j.1365-2788.2002.00408.x 12000585

[B55] Felce D Jones E Lowe K Perry J Rational resourcing and productivity: Relationships among staff input, resident characteristics and group home quality Am J Ment Retard 2003 108 161 172 10.1352/0895-8017(2003)108<0161:RRAPRA>2.0.CO;2 12691595

[B56] Down R Willner P Watts L Griffiths J Anger management groups for adolescents: A pilot study using quantitative and qualitative methods Clinical Child Psychology and Psychiatry 2011 16 33 52 10.1177/1359104509341448 20223794

